# Glycosaminoglycans from Alzheimer’s disease hippocampus have altered capacities to bind and regulate growth factors activities and to bind tau

**DOI:** 10.1371/journal.pone.0209573

**Published:** 2019-01-04

**Authors:** Minh Bao Huynh, Mohand Ouidir Ouidja, Sandrine Chantepie, Gilles Carpentier, Auriane Maïza, Ganlin Zhang, Joao Vilares, Rita Raisman-Vozari, Dulce Papy-Garcia

**Affiliations:** 1 Cell Growth, Tissue Repair and Regeneration (CRRET), UPEC EA 4397/ERL CNRS 9215, Université Paris Est Créteil, Université Paris Est, Créteil, France; 2 Aging and Neurodegenerative Diseases Brain Bank Investigation Laboratory, Universidade Federal de São Paulo, São Paulo, Brazil; 3 Université Pierre et Marie Curie-Paris 6, Centre de Recherche de l’Institut du Cerveau et de la Moelle Epinière (CRICM), Paris, France; New York State Institute for Basic Research, UNITED STATES

## Abstract

Glycosaminoglycans (GAGs), including heparan sulfates and chondroitin sulfates, are major components of the extracellular matrix. Upon interacting with heparin binding growth factors (HBGF), GAGs participate to the maintaintenance of tissue homeostasis and contribute to self-healing. Although several processes regulated by HBGF are altered in Alzheimer’s disease, it is unknown whether the brain GAG capacities to bind and regulate the function of HBGF or of other heparin binding proteins, as tau, are modified in this disease. Here, we show that total sulfated GAGs from hippocampus of Alzheimer’s disease have altered capacities to bind and potentiate the activities of growth factors including FGF-2, VEGF, and BDNF while their capacity to bind to tau is remarkable increased. Alterations of GAG structures and capacities to interact with and regulate the activity of heparin binding proteins might contribute to impaired tissue homeostasis in the Alzheimer’s disease brain.

## Introduction

Alzheimer’s disease (AD), the major dementia disorder in the aging population, is characterized by a slow but irremediably evolving degeneration of the brain tissue. Neuropathological analyses of the AD brain has shown a typical accumulation of extracellular senile plaques, composed of amyloid beta peptides, and intracellular neurofibrillary tangles (NFTs), made of the microtubule-associated protein tau [1]. In addition to the influence of these protein aggregates in the disease development, numerous researches in the domain aim to understand how the brain extracellular matrix (ECM) can affect cells capacities to growth, survive, and assure plasticity [2]. Overall, these cellular processes are regulated from the ECM by several growth factors, most of them belonging to the family of ‘heparin binding proteins’ (HBP) and so called ‘heparin binding growth factors’ (HBGF) [3, 4]. In general, the biological functions of HBGF rely on their capacities to interact with proteoglycans in the ECM, and particularly with their glycosaminoglycan (GAG) chains, which include heparan sulfates (HS) and chondroitin sulfates (CS) [5]. In brain, several HBGF including fibroblast growth factor 1 and 2 (FGF-1 and 2), brain derived neurotrophic factor (BDNF), heparin-binding epidermal growth factor-like growth factor (HB-EGF), vascular endothelial growth factor (VEGF) and pleiotrophin (PTN), exert their activities through interactions with GAGs [5]. FGF-2 mediates neurogenesis and neuronal survival [6, 7], BDNF is centrally involved in synaptic transmission and long-term synaptic plasticity [8], HB-EGF is a physiologic ligand for the EGF receptor (ErbB1) that importantly contribute to neuronal survival [9], and VEGF is a potent neurotrophic, neuroprotective, anti-apoptotic, and mitogenic HBGF [10] involved in the impaired angiogenesis observed in AD [11]. In addition, GAGs in the brain ECM interact with other proteins including and the microtubule associated protein tau [12], a HBP of central importance in AD [13]. Although classically considered intracellular, recent work in the field has shown that tau aggregates circulate in the extracellular space, although it is not clear whether extracellular tau accumulates or transiently accesses the extracellular space [14, 15].

In a previous work, we showed that sulfated GAG capacities to bind and potentiate HBGF activities are altered in the human aging hippocampus [16], a brain region controlling memory and cognition, and centrally affected in AD. However, it is unknown whether GAG levels and capacities to bind and potentiate HBGFs activities can be altered in hippocampus of AD. Here, we examined this question by extracting and analyzing AD hippocampal GAG levels and functionalities related to their capacities to interact and modulate the activity of some HBGFs. To consider the global sulfated glycosaminoglycanic complexity between the ECM in this tissue, we investigated AD and control total sulfated hippocampal GAG capacities to bind FGF-1, FGF-2, BDNF, VEGF, HB-EGF, and PTN. In order to get insights into GAG functional alterations, we studied the extracted GAG abilities to modulate the mitogenic activity of FGF-2 and VEGF, and the neuritogenic activity of BDNF. Additionally, we evaluated the AD and control hippocampal GAG capacities to interact with tau. To get structural insights on sulfation alterations in the matrix environment, we analyzed transcript levels of HS sulfotransferases, the family of enzymes conferring the largest degree of diversity to sulfated GAGs in cells and tissues. Our results indicate that the sulfated glycosaminoglycanic component of the ECM is structurally and functionally altered in hippocampus of AD, suggesting the implication of the brain glycanic matrix in the loss of tissue homeostasis in this disease.

## Materials and methods

### Tissue

Postmortem human hippocampus samples were from the Aging and Neurodegenerative Diseases Brain Bank Laboratory from São Paulo, Brazil. Subjects included in the study received postmortem evaluation by a board-certified neuropathologist. Two experimental groups were included (Table 1), a control (*n* = 5) and an AD group (*n* = 5) with subjects ages ranging from 64 to 84 years with a mean of 72.3 ± 7.0 years (s.d.). Post-mortem intervals averaged 14 h 33 min ± 6 h 18 min (s.d.). Protocols were approved by the local ethics committee ‘Comitê de Ética em Pesquisa do Hospital Universitário da Universidade Federal de São Paulo/Hospital de São Paulo (Unifesp/HSP-HU)’ with project number No. 285/04. Participant’s consent was verbally obtained from next-of-kin.

**Table 1:** Characteristics of control and AD subjects providing brain tissue.

Sex	Age (years)	PMD* (h)	Group	Immediate cause of death^†^	Senile plaques^‡^ by mm^2^	Braak and Braak^§^
M	65	8.2	Control	Hemotorax trauma	47	0
M	65	8.6	Control	Gun shot	51	I
F	73	15.3	Control	Brochopneumonia	65	II
F	64	14.3	Control	Liver traumatic rupture	41	I
F	76	24.0	Control	Myocardial infarction	65	I
*Mean* *± s*.*d*.	*68*.*6* *± 4*.*9*	*14*.*08* *± 5*.*7*				
M	84	19.0	AD	Bronchopneumonia	78	IV
F	70	10.3	AD	Bronchopneumonia	65	III
F	75	19.2	AD	Pulmonar trombosis	80	IV
F	69	5.4	AD	Myocardial infarction	76	IV
M	82	21.2	AD	Myocardial infarction	80	III
*Mean* *± s*.*d*.	*76*.*0* *± 6*.*1*	*15*.*1* *± 6*.*1*				

* PMD: Post mortem delay.

^†^ Subjects died from gunshot diagnosis did not show traumatic brain lesions.

^‡^ Senile plaques and NFTs values represent an arithmetic mean (mean ± s.e.m.) calculated from the counts of six microscopic fields for each observed region.

^§^ Braak and Braak stage.

Brains were obtained through autopsy and halved sagittally within 2 h after autopsy. One hemisphere was cut into 2-cm-thick slabs along the frontal plane and the hippocampus was dissected. Tissues were immediately frozen and stored at -80°C. Neuropathologic changes were investigated by following the Consortium to Establish a Registry for Alzheimer’s Disease and Braak and Braak guidelines. Senile plaques and NFTs were determined on Bielschowski-stained sections of middle frontal gyrus, middle temporal gyrus, inferior parietal lobule, occipital pole, hippocampal CA1 and entorhinal cortex. Senile plaques were counted using a 10x objective and NFTs were counted with a 20x objective.

### Glycosaminoglycans extraction and quantification

GAGs were extracted and quantified in the brain tissue as in [16, 17], with some modifications. Details on the extraction and quantification method validation (linearity, repeatability, reproducibility and analytical yield) are detailed in S1 File. Briefly, frozen hippocampus samples were homogenized and suspended in a buffer (50 mM Tris, pH 7.9, 10 mM NaCl, 3 mM MgCl_2_ and 1% Triton X-100) at 4°C. Samples were then treated with proteinase K to digest proteins (5 μg/mL; Merck) at 56°C overnight followed by heat-inactivation at 90°C for 30 min. DNAse (7.5 mU/mL; Qiagen) was added to samples to digest DNA and samples were incubated overnight at 37°C. Samples were brought to 2 M NaCl to avoid interaction between peptides, generated by PK digestion, with the sulfated GAGs. Then, peptides were eliminated by precipitation with trifluoroacetic acid (TCA, final 10%) at 4°C followed by centrifugation (13 000 g, 20 min). Supernatants were washed with chloroform (x2) to clear TCA and lipids, followed by dialysis to eliminate residual peptides from PK digestion, oligonucleotides from DNAse digestion, and many other small molecules (Slide-A-Lyzer Mini Dialysis Units 3 500 MWCO; Pierce); dialysis was carried against the buffer and then against water. After freeze drying, samples were dissolved in water or in a glycanase digestion buffer (10 mM CH_3_COONa, 2 mM CaCl_2_, pH 7), as required. Extracted GAGs were quantified with the 1,9-dimethyl-methylene blue (DMMB) assay [17]. Chondroitinase ABC (Chase ABC; Sigma-Aldrich) or nitrous acid treatment, were used to selectively quantify HS or CS in the extracted GAG samples as previously reported [16] and as in S1 File. Briefly, for HS quantification, total GAGs (2–3 μg) in 100 μL of a glycanase digestion buffer (50 mM CH_3_COONa, 2 mM CaCl_2_) were treated by Chase ABC (30 mU; Sigma-Aldrich) at 37°C for 1 h to eliminate CS in the samples. A CS spiked sample was used as control of ChaseABC activity. An additional Keratanase (30 mU; Sigma-Aldrich) treatment performed in some samples showed non-measurable levels of keratan sulfate (KS) in the brain tissue samples (not shown). After CS digestion, the residual HS were determined by following the DMMB protocol as described above. In each HS quantification experiment, a standard calibration curve was proceeded in the same way with known amounts of HS at concentrations ranging from 2.5–50 μg/mL (corresponding to 0.25 to 5 μg in the assayed samples). For CS quantification total sulfated GAGs were chemically digested by nitrous acid treatment as previously described [17] (S1 File). Briefly, 3 μg of total GAGs were diluted in 100 μL of H_2_O and mixed with 100 μL of sodium nitrite (NaNO_2_; Sigma-Aldrich) followed by addition of 100 μL of acetic acid (33%; VWR). Samples were incubated at rt for 1 h and the reaction was stopped by adding 100 μL of ammonium sulfamate (14%; Sigma-Aldrich). The CS remaining in samples were quantified by the DMMB protocol as described above. In each experiment, a CS-A calibration curve was proceeded in the same way with known amounts of standard CS-A (Sigma-Aldrich) at concentrations ranging from 2.5–50 μg/mL (corresponding to 0.25 to 5 μg in the assayed samples). A HS spiked sample was used for controlling nitrous acid digestion.

### Immunostaining

Tissue sections (20 μm) from AD and control brains were fixed with 3% acetic acid for 10 min at rt. Sections were incubated with 3% BSA dissolved in phosphate-buffered saline (PBS) and permeabilized (0.2% Triton X-100/PBS). HS were stained with an anti-HS (10E4, 1:200; AMS Biotechnology,) revealed with an Alexa-555 antibody (Invitrogen), followed by DAPI-labelling (1 μg/mL). Antibodies references are listed in S1 Table. Images were first obtained with a CCD camera (CFW-1310M, Scion Corporation, USA) in a BH-2 epi-fluorescence optical microscope (Olympus). Image acquisition was made by the Scion VisiCapture 2.0 software and processed by using ImageJ. DAPI labelling of nuclei was quantified as previously [18].

### GAG competition towards HBGF and tau

The capacities of GAG extracts to bind to human recombinant FGF-1, FGF-2, VEGF_165_, HB EGF (R&D systems), PTN and tau (Sigma-Aldrich) were evaluated by an ELISA based competition binding assay [16]. ELISA type 96 wells plates were coated with 2 μg/mL BSA-heparin conjugate solution prepared as in [19]. After washing with 0.05% Tween-20/PBS, wells were saturated with 3% BSA/PBS. Then, proteins (in PBS) were separately added to the wells in a concentration-response manner (0.1, 1, 10, 50, 100 and 500 ng/mL) to determine the concentration giving 50% of binding to immobilized heparin (ED_50_). Protein doses were fixed at 50 ng/mL (2.5 ng/well) for FGF-1, FGF-2, HB-EGF, PTN and 180 ng/mL (9 ng/well) for VEGF. Tau protein ED_50_ was fixed at 100 ng/mL (5 ng/well). These doses were used to determine the extent of protein binding to immobilized heparin in the presence of soluble competing GAG extracts. BSA was used as a negative control for a protein that does not bind to heparin (S1 Fig). Proteins and GAG extracts (at 0; 0.001; 0.01; 0.1; 1; 10; 100; 1 000 ng/mL in PBS) were simultaneously added to wells and incubated for 1 h at rt. Dextran 40 (Sigma Aldrich) at 1 000 ng/mL was used as negative control. After washing, the residual protein bound to the heparin conjugate was targeted by a corresponding antibody (1:1000, 1 h, rt) followed by a peroxidase-labelled secondary antibody (1:5000, 1 h, rt). The peroxidase activity was measured by the tetramethylbenzidine (TMB) detection kit (Pierce) and correlated to the amount of protein in the plate determined by an HBP calibration curve (0.1, 1, 10, 50, 100, 250 and 500 ng/mL). The amount of HBP bound to heparin decreased in the presence of soluble GAGs, which competed for the protein binding. Basal signal was assigned to 0% loss of signal in the absence of competitor, corresponding to 100% of HBP bound to immobilized heparin. A reference GAG-protein binding (100%) was assigned to the loss of signal observed when GAGs from control individuals were used as competitors. IC_50_ stands for the GAG concentration necessary to inhibit 50% of growth factor or tau binding to immobilized heparin. Antibodies and proteins references are listed in S1 Table.

### FGF2/GAGs dependent mitogenic activity

BaF32 cells [20], which require addition of exogenous GAGs to growth, were used to compare the capacities of AD and control hippocampus GAG extracts to induce FGF-2 dependent mitogenicity. Cells were cultured in RPMI 1640 (Invitrogen) supplemented with 10% fetal bovine serum (FBS) and 1 ng/mL interleukin-3 at 37°C, 5% CO_2_. 50 000 cells/well were cultured in RPMI medium supplemented with 10% horse serum. Under these conditions, FGF-2 dose-response (0.25 to 50 ng/mL) was first performed using heparin (2 μg/mL) as exogenous stimulator. An FGF-2 dose of 5 ng/mL, which gave 50% of response (FGF-2 ED_50_), was selected for further experiments. GAG extracts were then added to cells instead of heparin at concentrations ranging from 0.001 to 10 μg/mL. Non-treated cells and dextran (10 μg/mL) treated cells were used as control. After 46 h at 37°C, cells were incubated in the presence of ^3^H-thymidine for 4 h and then harvested onto a glass microfibre filter (Whatman Laboratory Division) using Automash 2000 Cell Harvester (Dynatech). Incorporated radioactivity was determined by scintillation counting on a 1450 Microbeta liquid scintillation counter (Wallac). Reference mitogenic response (100%) was assigned to the non-saturating mitogenic response, obtained from cells stimulated with FGF-2 (5 ng/mL) and control hippocampal GAGs (1 μg/mL).

### VEGF/GAGs dependent mitogenic activity

Differences on capacities of the hippocampal GAGs to induce human recombinant VEGF_165_ dependent mitogenic activity were examined in human umbilical vein endothelial cells (HUVECs). Cells were cultured at 37°C, 5% CO_2_ in complete EGM-2 BulletKit medium (Lonza) prepared from EBM-2 supplemented with 2% FBS and the complete set of supplied factors according to the manufacturer’s instructions. 30 000 cells/well were starved during 24 h with EBM-2 medium containing 0.5% FBS, ascorbic acid, gentamicin, amphotericin B (GA) and hydrocortisone [21]. VEGF_165_ (3 ng/mL) was then added alone or in combination with the hippocampal GAG extracts (from 0.03 to 33 ng/mL). Non-treated cells and dextran (33 ng/mL) treated cells were used as negative controls. After 24 h incubation at 37°C, 5% CO_2_, phase contrast images were taken by an Axiovert 10 microscope (Zeiss). Cells were fixed with ethanol and cell densities were evaluated by crystal violet signal cell count by means of calibration curves as in [21].

### BDNF/GAGs dependent differentiation

Hippocampal GAG capacities to potentiate human recombinant BDNF neuritogenic activity were analyzed in human neuroblastoma SH-SY5Y cells cultured in Dulbecco’s modified eagle medium (DMEM) supplemented with 10% FBS at 37°C. Cells (40 000 cells/well) were seeded and incubated for 24 h followed by a 24 h treatment with sodium chlorate (75 mM) to inhibit endogenous GAGs sulfation. BDNF (R&D Systems) was added at 200 ng/mL (10 ng/well) together with GAGs (1, 5 and 10 μg/well) and incubated for 48 h. Then, cells were fixed with 4% paraformaldehyde and permeabilized with 0.2% Tween 20 in 10% FBS/PBS for 1 h. Cells were stained with β-tubulin III antibody overnight at 4°C followed by anti-mouse IgG fragment-Cy3-fluorescent antibody. Images were obtained with a Nikon TE200 microscope coupled to a Hamamatsu CCD camera. Neurites length was measured by the NeuronJ plugin of the ImageJ software (https://imagescience.org/meijering/software/neuronj/). About 200 images were analyzed by group. Protein references are listed in S1 Table. Results are given as mean of neurite length by number of measured cells.

### RNA extraction and qPCR

Expression of HS sulfotransferases was evaluated by quantitative PCR (qPCR) in frozen homogenized powered hippocampus (CA1) samples as previously described [16]. Briefly, 1 mL of Trizole (Invitrogen) was added to 20–50 mg of frozen homogenized powered tissues. Chloroform (200 μL) was added and samples were stored for 5 min at 4°C. Aqueous phase was recovered and 0.5 mL isopropanol was added, mixed for 10 min at rt and centrifugated at 12 000 rpm, followed by sample pellet washing with 70% ethanol. Pellets were dried and dissolved in 40 μL of pure water. Samples were treated by DNase according to DNA-free Kit manufacturer’s instructions (Ambion). After purification, the amount of RNA was measured spectrophotometrically at 260 and 280 nm. The quality of RNA was confirmed by gel electrophoresis and by determination of the RNA Integrity Number (RIN) obtained by the ‘Standardization of RNA Quality Control Protocol’ in a 2100 bioanalyzer (Agilent) as in [16]. RIN was >7.5 average for all samples. Extracted RNA was used to synthesize complementary DNA (cDNA) by a reverse transcriptase reaction. Briefly, 1 μg of total extracted RNA was incubated with random primers (30 μg/mL) in a mixture of 5 mM dNTP’s and RNase inhibitor (Invitrogen) for 5 min at 65°C. Samples were then incubated with 1 mM DTT, RNase inhibitor and the Superscript II RNase H- Reverse Transcriptase (Invitrogen) for 52 min at 42°C and 15 min at 70°C. A mixture excluding the transcriptase served as a negative control. Genes of interest were analyzed in template cDNA by quantitative real time polymerase chain reaction (qPCR) using primers (Eurofins, Gemany) designed by Primer3output (S2 Table). qPCR was performed according to the LightCycler FastSart DNA Master SYBR Green kit manufacturer’s instructions (Roche). Relative gene expression was measured using the comparative CT method, also referred to as the ΔΔCT method [22]. Two housekeeping genes (*TUBA1A* and *TBP*) were used as endogenous controls. Normalization of these genes was accomplished with the Genorm program [23].

### Statistical analysis

Values are expressed as mean ± s.e.m or s.d. when indicated. The statistical significance of differences between the two groups was determined by *t*-test or one-way analysis of variance (ANOVA), as required, using the GraphPad Prism 5 software. Each sample was analyzed three times and each analysis was repeated three times. A *p* value < 0.05 was considered statistically significant. Note that * *p* ≤ 0.05, ** *p* ≤ 0.01 and *** *p* ≤ 0.001.

## Results

### GAG levels are altered in AD hippocampus

**Fig 1:**
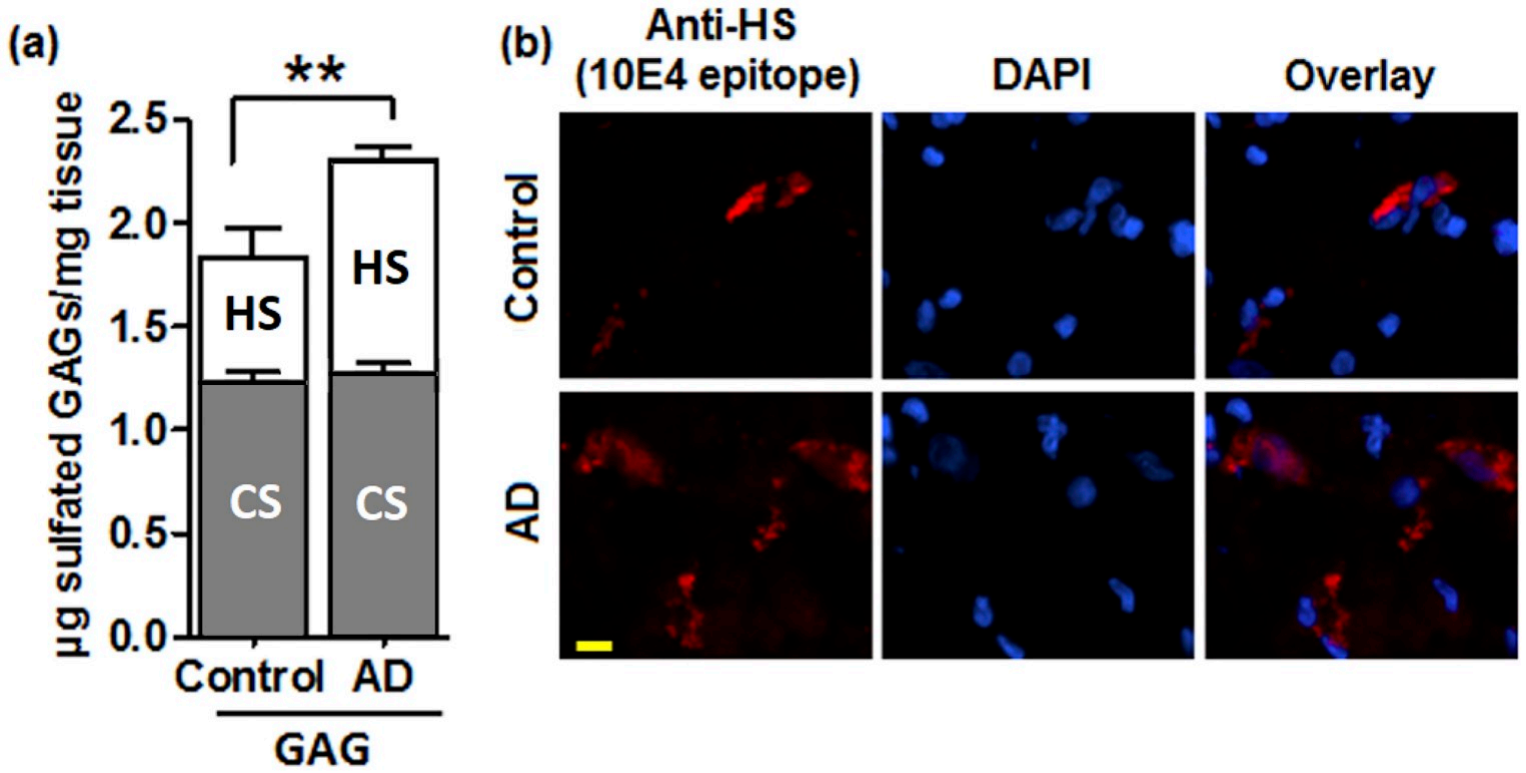
Sulfated GAGs levels are increased in AD hippocampus. **a)** GAGs were extracted from AD and control hippocampus and quantified by the DMMB method. HS and CS levels were measured after Chase ABC or nitrous acid digestion. Values represent mean ± s.e.m. (*n* = 5); t-test was used for statistical analysis, ** *p* ≤ 0.01. **b)** Immunostaining of HS was performed with the 10E4 antibody. Cell nuclei were stained by DAPI. Scale bar 10 μm.

Under physiologic conditions, the coordinated interaction of ECM macromolecules, including growth factors and sulfated GAGs, allows the maintain of brain tissue homeostasis [4]. To investigate whether endogenous sulfated GAG levels are altered in AD, we first extracted and quantified the total sulfated GAGs from AD and control hippocampus (*n* = 5). Our results show that sulfated GAG levels (Fig 1a) are significantly increased (*p* = 0.0089) in AD hippocampus (2.31 ± 0.06 μg/mg) compared to control tissue (1.84 ± 0.14 μg/mg). The GAG identities were confirmed by treating the extracted GAG samples with Chase ABC followed by nitrous acid, which respectively digests CS and HS. This completely suppressed the sulfated GAGs signal, indicating that CS and HS are the major sulfated GAGs in hippocampus. Accordingly, sulfated GAGs quantification remained unaltered in the keratanase treated samples (not shown). Moreover, selective HS and CS quantification indicate that HS are the GAG species that increase in the AD hippocampus, in accord with previous studies showing HS strong accumulation in the AD brain [12, 24–26] and as confirmed by the stronger HS immunostaining observed in AD hippocampus cryo-sections compared to control (Fig 1b).

### AD hippocampal GAGs altered capacities to bind growth factors

Since HBGF functions are mediated through their interactions with GAGs, we analyzed the extracted hippocampal GAG capacities to bind to some growth factors involved in brain tissue homeostasis. We used an ELISA competition assay to investigate whether GAG capacities to bind FGF-1, FGF-2, VEGF_165_, HB-EGF and PTN are altered in AD. In this assay, the different soluble GAGs can be compared for their capacities to better interact with a given protein, the better the interaction is with the competitor GAG, the lower the protein is found to bind to the immobilized heparin [16, 19]. Our results in Table 2 and Supplementary Fig (S2 Fig) indicate that, compared to GAGs from control individuals, GAGs from AD shows decreased capacities to bind to FGF-1 (*p* = 0.0087), FGF-2 (*p* = 0.0014) and VEGF_165_ (*p* = 0.0051). In contrast, the AD GAGs binding to HB-EGF and PTN capacities were significantly increased (*p* = 0.0003 for HB-EGF and *p* = 0.0025 for PTN).

**Table 2:** Hippocampal AD and control GAGs relative binding to growth factors.

Growth factor	Hippocampal GAGs IC_50_^a^ (ng/mL)		AD *vs* control (folds)
	Control (*n* = 5)	AD (*n* = 5)	
FGF-1	8.0 ± 1.2	29.8 ± 6.0	3.8 ↑ **
FGF-2	61.6 ± 2.6	86.6 ± 4.4	1.4 ↑ **
HB-EGF	100.2 ± 9.8	25.8 ± 7.4	3.8 ↓ ***
PTN	27.8 ± 6.4	10.2 ± 2.0	2.7 ↓ **
VEGF_165_	29.0 ± 5.6	81.4 ± 9.0	2.8 ↑ ***

IC_50_: Effective concentrations required to inhibit 50% of binding of the growth factor to immobilized heparin. IC_50_ was calculated as described in Materials and Methods from data presented in S2 Fig.

** *p* ≤ 0.01,

*** *p* ≤ 0.001 (*t*-test of control *vs* AD group); the arrow before starts indicates increased or decreased IC_50_.

### AD GAGs altered capacities to bind and potentiate HBGF

**Fig 2:**
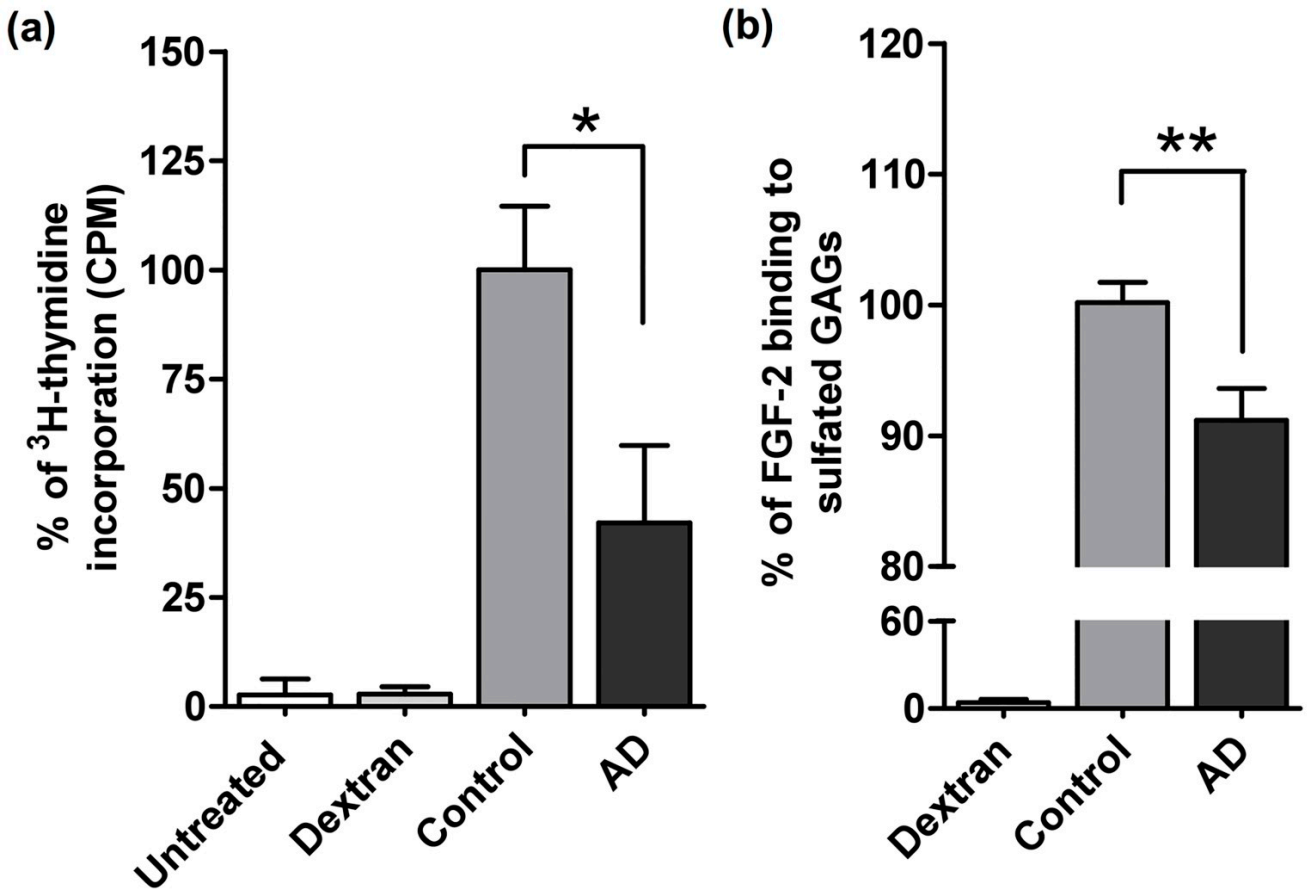
GAGs from AD hippocampus show lower capacities to bind FGF-2 and to potentiate its mitogenic activity. **a)** GAG dependent FGF-2 mitogenic activity was assayed on BaF32 cells and detected by ^3^H-thymidine incorporation after stimulation with AD and control hippocampal GAGs. The reference effect (100%) was assigned to the FGF-2 (5 ng/mL) dependent mitogenic response obtained from cells treated with 1 μg/mL of control GAGs (determined by dose-response experiments). Cells non-supplemented with GAGs or supplemented with dextran were used as negative controls. **b)** AD *vs* control hippocampal GAGs relative binding to FGF-2 assessed by the ELISA competition assay in where extracted GAGs (50 ng/mL) competed with immobilized heparin to bind the growth factor (50 ng/mL). A reference GAG-protein binding (100%) was assigned to the competitive loss of signal obtained with the control GAGs. Basal signal was assigned to the 0% competition in the absence of competitor. The amount of protein in the heparin-immobilized plate was determined by using a calibration curve. Values represent mean ± s.e.m. (*n* = 5); one-way ANOVA was used to determine significance, * *p* ≤ 0.05 and ** *p* ≤ 0.01.

It is known that GAG capacities to regulate cell functions depend on their abilities to bind to the different HBGF and to modulate their activities [27]. To examine whether the hippocampal sulfated GAG functionalities are altered in AD, we tested their capacities to potentiate the mitogenic activities of FGF-2 and VEGF, as well as the neuritogenic activity of BDNF, in cultured cells. The mitogenic effect was first examined in BaF32 cells, a lymphoblastoid cell line devoided of cell surface HS and overexpressing the FGF receptor type 1 (FGFR1). These cells respond to FGF-2 only if GAGs are added to cell culture medium [20]. In this system, the control AD’s GAGs extracts showed to induce significantly lower mitogenic activity than GAGs extracted from control hippocampus (Fig 2a). This is in accord with the decreased capacities of AD GAGs to bind to FGF-2 (Fig 2b) and translating a low capacity of the AD GAGs to potentiate FGF-2 activity in cells. As expected, cells non-supplemented with GAGs, or supplemented with dextran, failed to proliferate.

**Fig 3:**
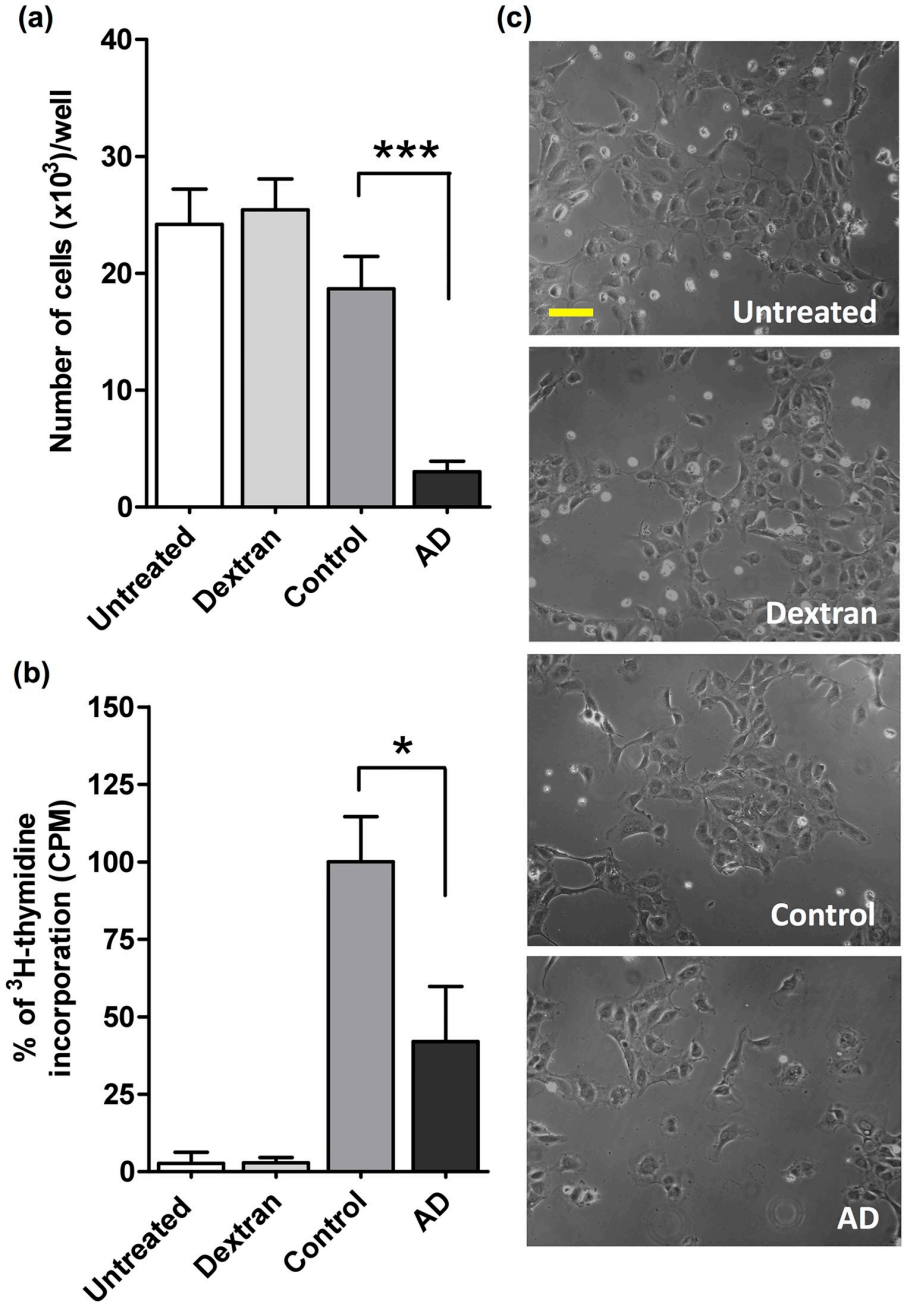
GAGs from AD hippocampus have lower capacities to bind VEGF and to potentiate its activity. **a)** VEGF_165_ induced HUVEC proliferation after stimulation of GAG extracts from AD and control hippocampus. VEGF_165_ (3 ng/mL) was added to cells in combination with GAG extracts (3 ng/mL). Cell densities were evaluated by crystal violet [21] after 24 h. The VEGF_165_ and GAG concentrations were determined by dose-response experiments. Cells non-supplemented with GAGs or supplemented with dextran were used as negative controls. **b)** AD *vs* control GAG relative binding to VEGF were analyzed by an ELISA competition assay. GAG extracts competed with immobilized heparin to bind VEGF_165_ (180 ng/mL). The signal was recorded when control hippocampal GAGs (50 ng/mL, determined by dose-response experiments) was considered as control reference (100%). **c)** Phase contrast images of HUVECs stimulated with extracted AD or control GAGs in the presence of VEGF_165_. Images were taken by an Axiovert 10 microscope (Zeiss). Scale bar: 50 μm. Values represent mean ± s.e.m. (*n* = 5); one-way ANOVA was used to determine significance, *** *p* ≤ 0.001.

We then tested the AD GAG capacities to induce HUVEC cells proliferation. Results in Fig 3a and 3c show that, in agreement with the VEGF_165_ binding experiments (Fig 3b), AD GAGs showed lower capacities to stimulate VEGF_165_-dependent growth of HUVEC cells when compared to control GAGs. This suggests that the AD GAGs have a decreased capacity to potentiate VEGF mitogenic activity.

**Fig 4:**
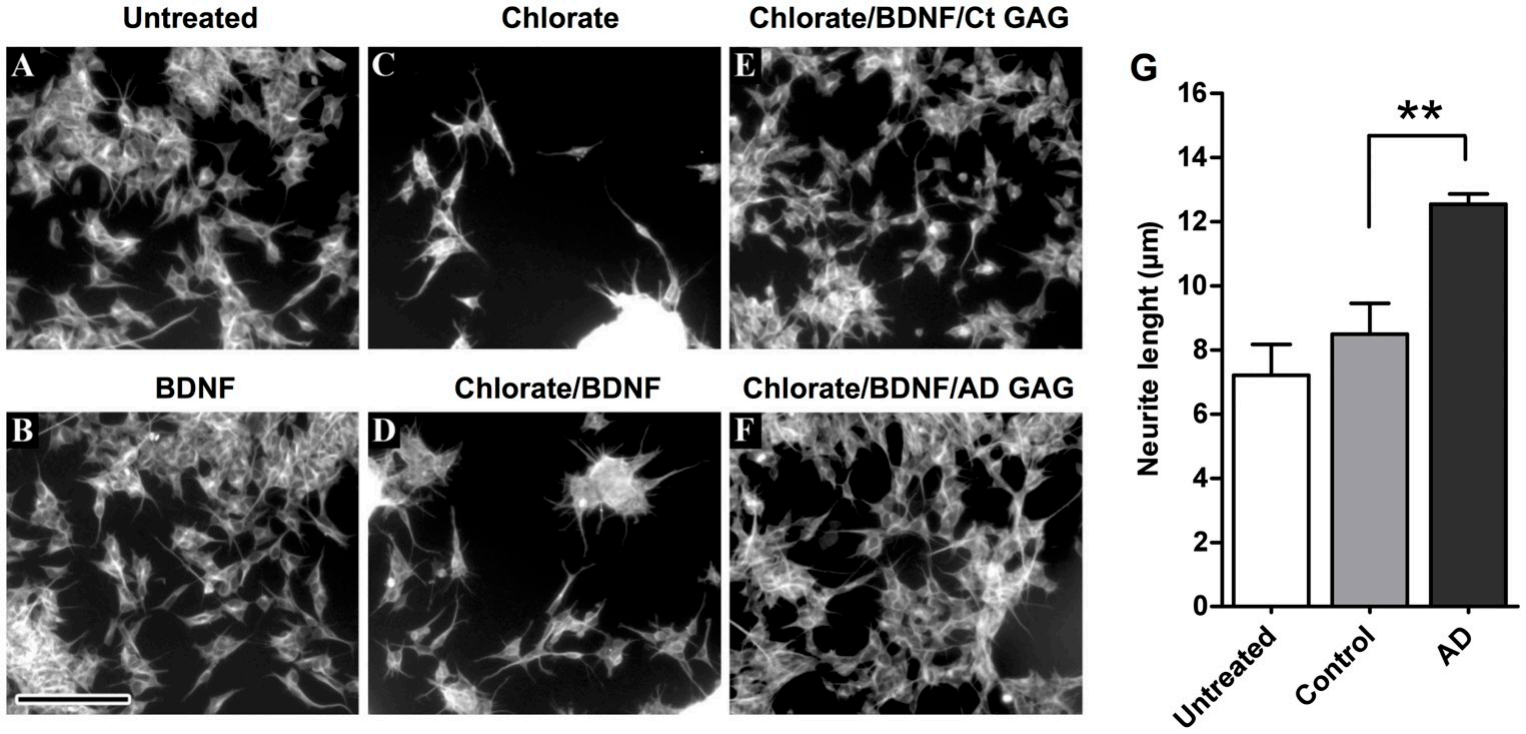
GAGs from AD hippocampus capacities to potentiate BDNF neuritogenic activity. SH-SY5Y cells were differentiated with sodium chlorate (75 nM) and stimulated with BDNF (200 ng/mL) or in combination with hippocampal GAGs (1 μg/mL). Sodium chlorate was used to inhibit endogenous GAGs sulfation. Sodium chlorate, BDNF, and GAG concentrations were fixed by dose-effect experiments. Fixed cells were permeabilized and stained by β-tubulin III. **a)** Control untreated SH-SY5Y cells. **b)** BDNF only treated cells. **c)** Sodium chlorate only treated cells. **d)** Sodium chlorate/BDNF co-treated cells. **e)** Sodium chlorate/BDNF/control GAGs co-treated cells. **f)** Sodium chlorate/BDNF/AD GAGs (1 μg/mL) co-treated cells. **g)** Neurogenic effect, expressed by neurite length, in BDNF/chlorate treated cells supplemented with AD or control GAGs. Image processing was done by measuring the neurite length (NeuronJ software). Scale bar: 50 μm. Zoom factor for the inset is 2X. Values represent mean ± s.e.m. (*n* = 5);, one-way ANOVA was used to determine significance,** *p* ≤ 0.01.

We then tested the AD GAG capacities to potentiate BDNF neuritogenic activity in neuroblastoma SH-SY5Y cells. To examine the effect of the hippocampal GAGs, we pre-treated cells with sodium chlorate, a known inhibitor of GAGs sulfation, to circumvent the cellular sulfated GAGs that mask the exogenous GAG effects. Accordingly, BDNF treated cells did not show any measurable effect compared to control cells (Fig 4a *vs* 4b), while the effect of suppressing the endogenous GAGs sulfation showed altered cell morphology, with cells forming clumps, as compared to control cells (Fig 4a *vs* 4c). The effect of BDNF was evidenced in the sulfated GAGs deficient cells since in cells stimulated with BDNF these clumps were reduced (Fig 4c *vs* 4d). Addition of extracted GAGs to the BDNF treated cells avoided the cell clumping phenomena and restored normal cell morphology (Fig 4d *vs* 4e and 4f), with a higher effect on the AD extracted GAGs compared to the control ones (Fig 4e *vs* 4f). Accordingly, image analysis of the BDNF-GAGs treated cells showed that AD GAGs induced a slight but significantly higher neuritogenic effect; average neurite length was 12.55 ± 0.32 μm/cell in AD GAGs stimulated cells while it was 8.50 ± 0.95 μm/cell in controls GAG stimulated cells (Fig 4g). Interestingly, the capacity of the extracted GAGs to compete for BDNF binding to heparin could not be assessed by the ELISA competition assay since the growth factor could not bind to heparin, suggesting that the BDNF effect observed in cells is related to GAGs structures not presented in heparin but present in the GAG extracts, opening to an interesting line of investigation. Taken together, these results show a clear difference on the AD GAG trophic activities compared to control GAGs.

### AD GAGs capacities to bind tau

**Fig 5:**
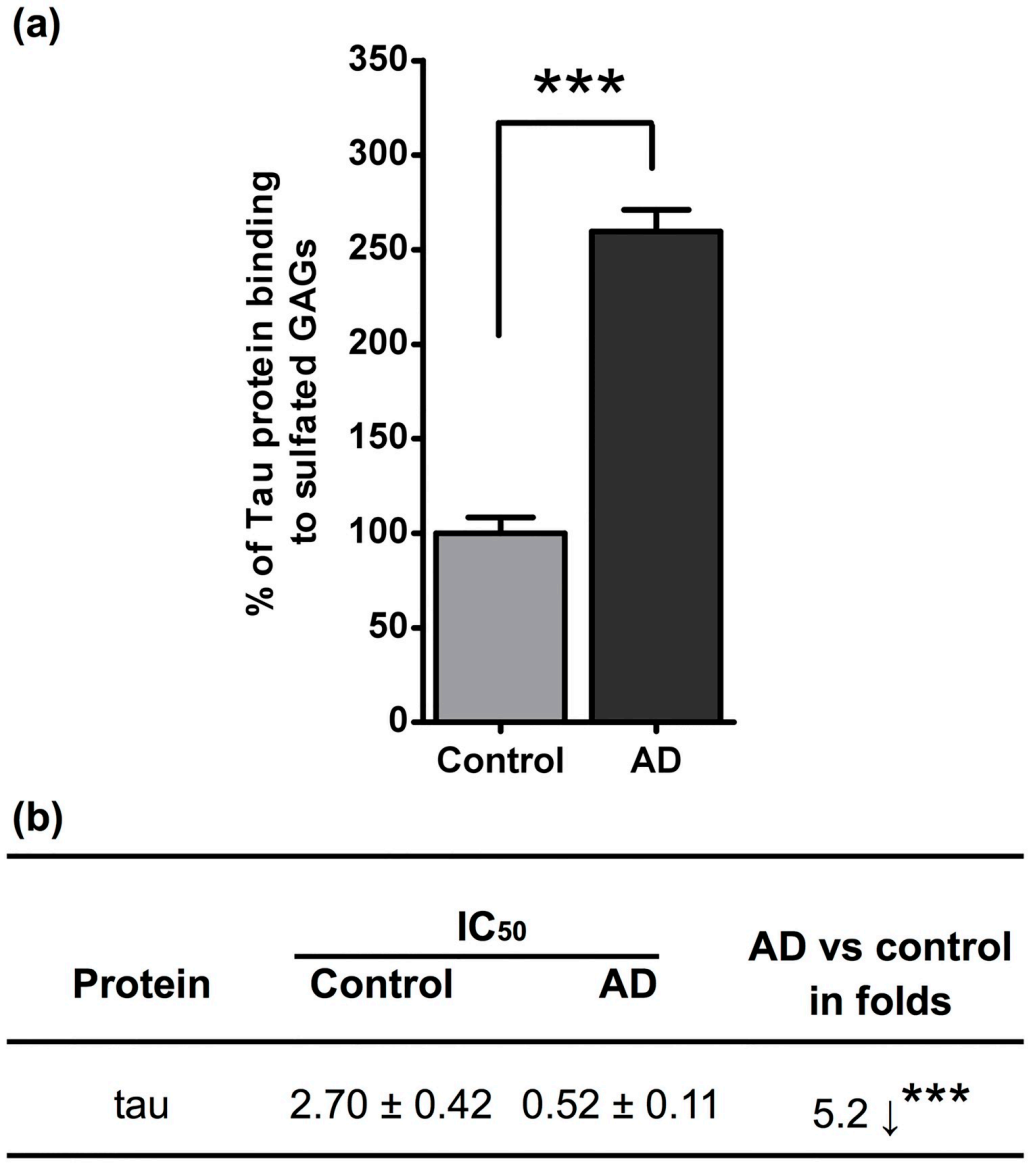
Sulfated GAGs from AD hippocampus show increased capacities to bind tau. **a)** Sulfated GAGs binding to tau was determined by the ELISA competition assay. Tau (100 ng/mL) and GAGs (0.1 ng/mL) concentrations were determined with dose-response experiments. The signal given by control GAGs was considered as 100% effect. **b)** IC_50_ changes in tau binding capacities with GAGs. IC_50_ stands for the GAG concentration necessary to inhibit 50% of tau binding to immobilized heparin. Values represent mean ± s.e.m. (*n* = 3); t-test was used to determine significance, ** *p* ≤ 0.01.

Tau is a HBP that accumulates with sulfated GAGs in AD brain and in cells models of tauopathy [24, 26, 28–31]. Here, we used the ELISA binding assay to compare the capacities of AD and control GAGs to bind to tau. We observed that GAGs extracted from the AD brain presented a significantly higher capacity to bind to tau compared to GAGs from control tissue (Fig 5a), as translated by the GAGs concentration necessary to inhibit 50% of tau binding to the immobilized heparin (Fig 5b). Interestingly, among the here studied HBP, tau was the one for which the AD GAGs showed the highest increased binding capacity.

### Altered expression of HS metabolic enzymes in AD hippocampus

**Fig 6:**
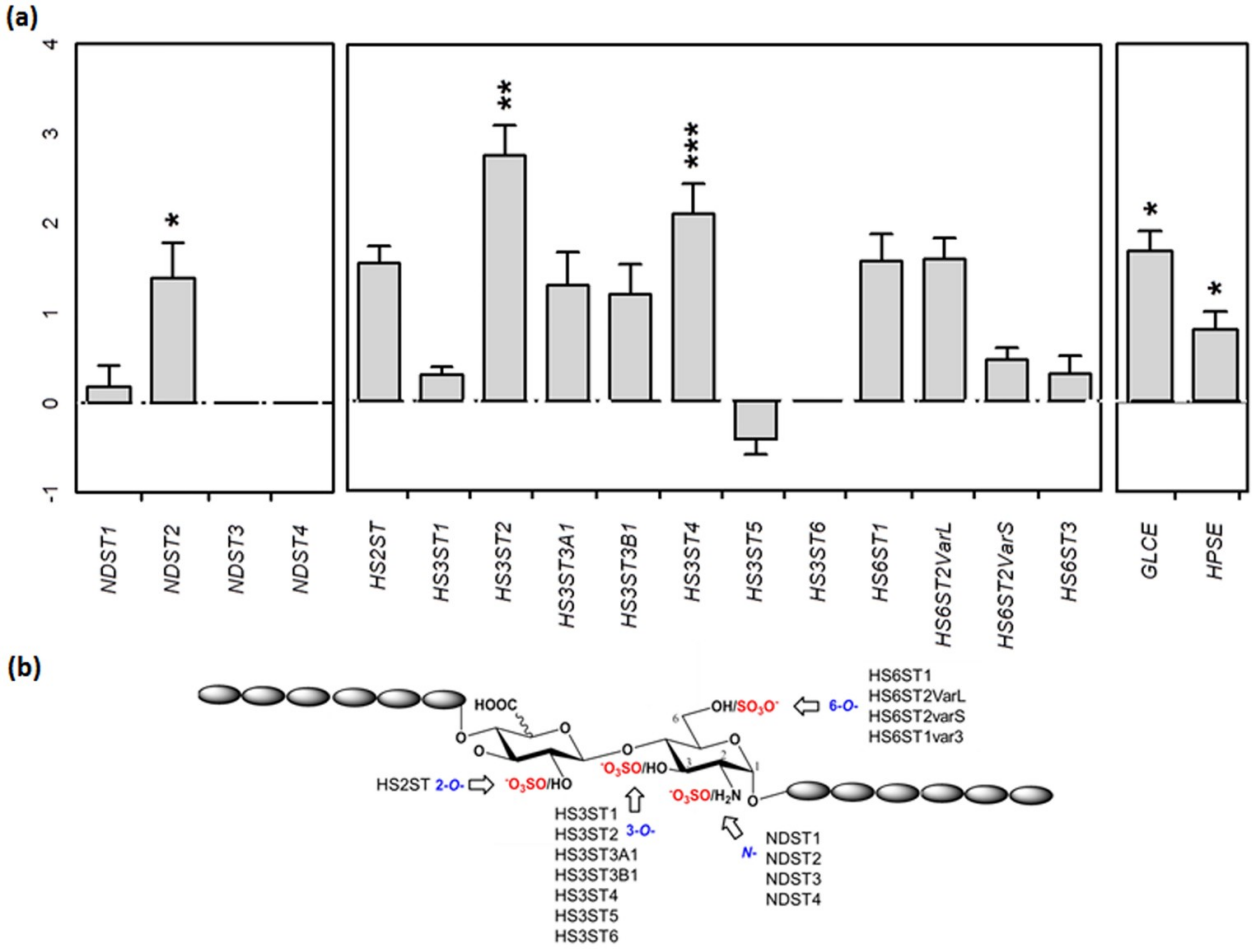
HS sulfotransferases are increased in AD hippocampus. **a)** The expression of the main HS sulfotransferases responsible of *N*-, 2-*O*-, 3-*O*- and 6-*O*-sulfation were examined by RTqPCR. Other genes were analyzed including C5-epimerase (*GLCE*), heparanase (*HPSE*) and carbohydrate sulfotransferase 8 (*CHST8*). Two reference genes (TUBA1A and TBP) were used as endogenous controls. Expression on control individuals was set to one to visualize over and down expressions. Significant change in transcript expression * *p* < 0.05 or ** *p* < 0.01. **b)** Sulfation positions of HS chains in a representative HS disaccharide. *N*-, 2-*O*-, 3-*O*-, and 6-*O*-sulfation of HS are respectively assured by *NDSTs*, *HS2STs*, *HS3STs* and *HS6STs*.

We showed that sulfated GAG levels, and particularly HS levels, are increased in the AD hippocampus and that the total sulfated GAG capacities to differentially bind to growth factors and tau are altered. Thus, to investigate possible modifications on the sulfation patterns of HS in AD, we analyzed transcript levels of enzymes responsible of sulfation of these structurally complex GAG species in the AD and control tissue. We used quantitative PCR (qPCR) to examined the expression of the main HS sulfotransferases responsible of *N*-, 2-*O*-, 3-*O*-and 6-*O*-sulfations in human: four *N*-deacetylase/*N*-sulfotransferases (*NDST1*, *2*, *3*, and *4*), one 2-*O*-sulfotransferase (*HS2ST*), seven 3-*O*-sulfotransferases (*HS3ST1*, *2*, *3A1*, *3B1*, *4*, *5* and *6*), and four 6-*O*-sulfotransferases (*HS6ST1*, *variant 2L*, *2S*, and *3*) together with C-5 epimerase (*GLCE*). Carbohydrate sulfotransferase 8 (*CHST8*) expression, previously reported to be altered in prion diseases [32, 33], was also examined. Two housekeeping genes (*TUBA1A* and *TBP*) were used for normalization with the Genorm program [23]. Our results show that most sulfotransferase transcripts were increased in AD brain compared to control tissue (Fig 6). However, significant overexpression was mainly observed for *HS3ST2* and *HS3ST4*, which are predominantly expressed in brain. These results suggest the existence of altered sulfation patterns in HS GAGs in the AD brain.

## Discussion

In all tissues, the ECM plays key roles in the regulation of tissue homeostasis by assuring GAG-driven growth factors interactions with their high affinity cellular receptors. These interactions rule out tissue integrity by offering to cells the necessary signals to growth, to differentiate and to survive [3, 4]. In order to investigate whether the sulfated GAG functionalities are altered in the AD brain, we studied GAG levels and capacities to interact and promote the activity of some HBP, including growth factors and tau. We extracted and quantified GAGs from AD and control hippocampus using an efficient method validated for brain tissue [16] (S1 File). GAG quantification showed a significant total sulfated GAGs increase in AD tissue, suggesting dysregulated tissue homeostasis. This increase in GAGs content might be the result of altered tissue homeostasis which might induce compensatory GAG synthesis in the disease tissue. Interestingly, selective digestion of HS *vs* CS indicated that the increase in total sulfated GAGs is related to an increased in HS levels, in accord with previous studies showing that HS, and not CS, are increased in the AD brain [26, 30, 34, 35]. This is also in accord with the characteristic patterns of tau protein deposition, which largely excludes the zones abundant in CS, as those rich in perineuronal nets [14, 34]. Nevertheless, more studies are required to clarify if GAGs are involved or not in the mechanisms leading to tau protein deposition in AD.

In order to investigate whether changes in GAG levels could be translated by changes in their physiological functionalities, we evaluated the extracted GAG capacities to bind to HBGFs known to be involved in the regulation of brain cells functions. Our results showed that the capacities of GAGs to bind FGF-1, FGF-2, VEGF, HB-EGF and PTN were altered in AD. Interestingly, while the AD GAG binding capacities decreased for FGF-1, FGF-2, and VEGF, they increased for HB-EGF and PTN, a HBGF, suggesting that GAG chain alterations differently modify their capacities to modulate the activity of growth factors. The differences observed on the AD GAG capacities to bind to the different growth factors, some of which increased while other decreased, are in accord with the proposed diversity of GAG structures and capacities to differently bind to proteins [5]. Indeed, different GAG chains can coexist in a same tissue, or even a single GAG chain could carry different sulfated sugar sequences, which could interact with different proteins coexisting in a tissue. Our results are in accord with this concept and show that binding to each protein can differently vary in disease. In agreement with a possible biological significance of the altered GAG capacities to interact with growth factors, AD GAG decreased affinities for FGF-2 and VEGF_165_ were confirmed by their decreased capacities to potentiate the growth factors mitogenic activity in cultured cells. FGF-2 shares many similarities with classic neurotrophins [36], it is implicated in neurogenesis, improves spinal cord injury, and has neuroprotective effect in motor neurons [37]. Moreover, the decreased binding of AD GAGs to FGF-1 is in accord with previous works in where FGF-1 acted as a neuropoietic mediator and promoter of neuron survival in brain [38, 39]. Likewise, the AD GAGs decreased VEGF binding and capacity to potentiate activity is in accord with the previously observed loss of VEGF capacity to promote brain angiogenesis, neuroprotection and cerebro-microvascular permeability in AD [40, 41]. Our results suggest that, in AD, the lower GAG capacities to bind and activate VEGF_165_ and FGF-2 might contribute to an altered angiogenic state, and probably to alterations of other neurotrophic processes [42], as neurogenic lineage activation, neuritogenesis and neural plasticity. On the other side, our result showed that GAGs from the AD hippocampus exhibit both an increased capacity to bind to BDNF and to induce its neuritogenic activity in neuro-differentiated SH-SY5Y cells. This is in accord with reports describing GAGs as mediators of synaptic plasticity promoted by BDNF through the tropomyosin-related kinase (Trk) B and p75 neurotrophin (NTR) receptors in mature neurons [8]. However, our results showed that BDNF could not bind to immobilized heparin, indicating that the here observed neuritogenic effect was related to GAG structures not present in heparin chains. Interestingly, the neurogenic effect was higher in cells stimulated with GAGs from AD than from control tissue, suggesting a that the sulfated GAGs could be involved in a trophic compensatory response in AD, although this remains to be explored. Taken together, our results suggest the existence of divergent alterations of growth factors activities that possibly participate to loss of homeostasis in the AD tissue. Moreover, increased GAG capacities to bind to tau suggest that the alteration of GAG structures might alongside affect processes characteristic of the AD pathology, as tauopathy. Indeed, a number of evidences have shown that sulfated GAGs are able to induce tau aggregation and to promote cell to cell tau proteopathic seeds propagation through the ECM [35, 43]. Since tau is known to interact with HS at both intracellular and extracellular levels [15, 28], intracellular HS can promote the monomeric tau abnormal phosphorylation [28, 44] and possibly aggregation [13], while cell-membrane associated HS can promote tau proteopathic seeds uptake by healthy recipient cells [15, 45]. Thus, it is possible to considered that enhanced HS-tau binding capacities might participate the extent of the tau biological fates in AD.

GAG-protein interactions are generally driven by sulfation levels and patterns in the GAG polysaccharidic chains. To investigate whether GAGs sulfation, and particularly HS sulfation, is altered in the AD tissue used in this study, we measured the transcripts levels of all human HS sulfotransferases. We used qPCR to examine the expression of sulfotransferases responsible of *N*-, 2-*O*-, 3-*O*-and 6-*O*-sulfation, together with other HS metabolic enzymes as heparanase (*HPSE*) and HS epimerase (*GLCE*). Our results showed that most sulfotransferase transcripts were significantly increased in the AD tissue, in accord with high HS sulfation in AD brain [29, 31]. Nevertheless, among the different sulfotransferases, a higher significant increase of expression was observed for *HS3ST2* and *HS3ST4*, enzymes responsible of 3-*O*-sulfation in brain [46]. 3-*O*-sulfation is a rare and minor sulfation in HS chains and although it might only account for a low global charge effect in HS chains, it might strongly affect HS properties, as recently proposed [28]. However, *HS3STs* are not the only increased sulfotransferase transcripts in the AD tissue, *NDST2*, *HS2ST*, and *HS6STs* are also increased. Similarly, epimerase and heparanase expressions are also increased, although at lower but significant extents, suggesting increased HS C-5 epimerization and HS catabolism in disease. Our data suggest that HS sulfotransferases, and particularly *HS3STs*, are overexpressed in AD. However, recent work showed that *HS6ST* might play an important role on tau binding during AD pathology [47, 48]. Moreover, we found that expression of *CHST8*, involved in sulfation of *N*-acetylgalactosamine (GalNAc) residues on *N*- and *O*- glycoproteins, and likely in CS chains, [32, 33], is also slightly increased. This opens to the possibility of a discrete and complex pattern of GAGs sulfation in AD that require to be explored by investigating the functional relevance of the different identified genes in an appropriate experimental system (e.g. via genetic manipulation in cells via knockout/ knockdown and functional readout of tau binding/ uptake or seeding). Concerning CS involvement, more experiments are still required to study a possible involvement of CS since studies have shown that CS are not accumulated in the AD brain [26, 30, 34]. Globally, our results together with the current literature in the domain, stand for structurally altered HS species in AD hippocampus with increased sulfation and changes in the GAG capacities to interact with heparin binding proteins including growth factors and tau. Interestingly, although there is few information about GAGs expression in the healthy brain and about their differential expression in different brain areas, it has been shown that the AD brain accumulates highly sulfated GAGs in regions affected by the tauopathy, as cortex and hippocampus [24, 25, 26], and not in cerebellum [31], a brain region not affected by the tau pathology in AD. Further studies to investigate GAG expression specificities on the different brain regions in healthy and AD brains are in progress, as well as investigations on the AD-GAGs capacity to interact and modulate the tau abnormal phosphorylation, aggregation, and spreading.

## Conclusion

Overall, our results show that sulfated GAGs are increased in AD hippocampus and that this increase is accompanied by changes in the GAG capacities to interact with growth factors and tau. Concerning the growth factors, these changes affect not only the sulfated GAG capacities to interact with these proteins, but also to modulate their activities, as shown here for FGF-2, VEGF_165_, and BDNF. This makes conceivable that altered GAG structures similarly influence the activity of therapeutic growth factors and cells. Taken together, these results advance our understanding of the significance of the glycanic ECM quality and functionality during disease. However, further research is still required to finely reveal the individual GAG types, structures and exact sulfated sequences in the GAGs chain. This might open to new concepts for therapeutic remodelling of the altered brain glycanic matrix in AD.

## Supporting information

S1 File Sulfated glycosaminoglycans extraction from brain tissue and quantification: Method validation. (PDF)

S1 Table List of recombinant proteins and antibodies. (DOC)

S2 Table List of oligonucleotides for real time qPCR. (DOC)

S1 Fig Control BSA binding on heparin. (TIF)

S2 Fig Hippocampal AD and control GAGs relative binding to growth factors. (TIF)
