# Supplementary material for: Glycosaminoglycans from Alzheimer’s disease hippocampus have altered capacities to bind and regulate growth factors activities and to bind tau
Source: PLoS One. 2019 Jan 4;14(1):e0209573. doi: 10.1371/journal.pone.0209573 (PMC6319808; doi:10.1371/journal.pone.0209573)
Supplement: S1 File — (PDF) [file pone.0209573.s001.pdf]

S1 File. Sulfated glycosaminoglycans extraction from brain tissue and quantification: Method validation

Minh Bao Huynh, Sandrine Chantepie, Dulce Papy-Garcia

METHODS

Products and equipment

All chemicals and biological products were of the best grade and used as supplied. Sodium formate, formic acid, and proteinase K were from Merck. Propan-1-ol was from Prolabo-VWR. All other chemicals, including chondroitin sulfate A (CS-A), heparan sulfate (HS), 1,9-dimethylmethylene blue (DMMB), BSA, were from Sigma-Aldrich. Ethidium bromide was from Interchim. DNase I was from Qiagen. TBE buffer was from Invitroge. Electrophoresis unit was from Sigma/Biorad and gel visualisation system Chemigenius from Syngene. Optical density was recorded in a microplate iEMS reader MF from Labosystems. For shaking of samples, a vortex-Genie with a plate (Scientific Industries) was used.

Tissue

The sulfated GAGs extraction and quantification method was validated in brains from four months old male rats (Janvier, France). European regulations for the care and use of laboratory animals were observed. Anaesthesia was induced by intraperitoneal injection of sodium pentobarbital (50 mg/kg). Total brains were dissected and frozen in liquid nitrogen, in some cases cortex was separately recovered. Before storing at -80°C, a part of tissue was freeze-dried. Tissue was then referred as cortex or total brain ‘wet tissue’ or ‘freeze-dried tissue’.

Total GAG extraction

Protein and DNA digestion step: Brain tissue, referred as cortex or total brain, wet and freeze-dyed, were weighted and suspended in an extraction buffer (50 mM Tris-HCl, 10 mM NaCl, 3 mM $MgCl_2$, pH 7.9) for final 25 mg tissue/mL suspension. Proteins were digested by proteinase K (PK) treatment (5 µg/mL) at 56 ºC overnight followed by 30 min incubation at 90 ºC. PK digested samples were submitted to DNA digestion or were spiked in triplicate with different concentrations of CS-A (to final 5, 10, 20 and 30 µg/mL concentrations) before DNA digestion for further method validation. PK digested samples (100 µL), spiked or not with CS-A, were treated by 7.5 U of DNase final concentration and samples were incubated overnight at 37 ºC. To asses DNAse digestion, samples were dissolved in electrophoresis loading buffer (25% phenol bromide blue, 25% xylene cyanol FF, 30% glycerol, 20% H_2O) and added into a 1% agarose gel prepared with 25 µg (one drop) of ethidium bromide in 1X TBE buffer which also was used as a running buffer. The running time was 45 min at 100 volts (60 mA). The gel was visualized with UV light.

Residual DNA elimination and lipids extraction step: PK and DNase treated samples were diluted 1:1 with 4 M NaCl and centrifuged (13 000 g, 20 min). Pellets from samples and form CS-A spiked samples were controlled to be free of GAG (by DMMB test as indicate below) and then discarded. Lipids were eliminated from samples by chloroform extraction (1:1 v/v). Total sulfated GAGs were then quantified as detailed below.

Detail on the preparation of CS-A spiked samples for method validation: Wet and freeze-dried PK digested cortex and total brain samples were spiked with a CS-A solution for 5, 10, 20 and 30 µg/mL final concentrations on the PK treated samples. Spiked brain samples were as follows: freeze-dried cortex, wet cortex, freeze-dried total brain and wet total brain. Fractions of 100 µL of spiked samples were used for the total GAGs extraction and quantification protocol. GAGs showed to be stable under the whole conditions.

Total sulfated GAG DMMB quantification

The total sulfated GAGs quantification procedure was accomplished according the 1,9-dimethylmethylene blue (DMMB) assay as previously described [1]. The method consists in a primary complexation of the total sulfated GAGs with the DMMB dye. The amount of DMMB complexed to GAGs is proportional to the amount of the sulfated GAGs in the sample. The formed GAG-DMMB complex becomes insoluble in the complexation conditions, and precipitates. After elimination of un-complexed DMMB dye that remained in solution, the GAG-DMMB complex (pellet) is washed and re-solubilized in a decomplexation solution. The absorbance of the resolubilized DMMB is the proportional to the amount of sulfated GAGs present in the sample.

Preparation of the GAG/DMMB complexation solution: A DMMB 1.85 mM ethanol solution was prepared and filtered (paper filtration). This solution was mixed (1:1 v/v) with a solution containing 0.2 M guanidine hydrochloride, 0.2% formic acid, and 0.2% sodium formate. The resulting complexation solution can be stored without deterioration for several months at room temperature in the darkness.

Preparation of the GAG/DMMB decomplexation solution: A solution made of 50 mM sodium acetate containing 4 M guinidine hydrochloride and 10% propan-1-ol (pH 6.8) was prepared. The solution can be stored for several months at room temperature without deterioration.

GAG quantification protocol: Typically, 100 µL of standards, samples, or spiked samples were used. The DMMB complexation solution (1 mL) was added each sample and samples were vigorously agitated for 30 min. The resulting GAG-DMMB complex suspension was centrifuged for 10 min (10 000 g) and the excess non-complexed DMMB was eliminated. Then, 250 µL of decomplexation solution was added and samples were again vigorously agitated and centrifuged. The amount of DMMB in solution, proportional to the amount of sulfated GAGs on samples, was measured at 656 nm in supernatants. A calibration curve

extemporaneously constructed with known amounts of CS-A standard was included in every assay. Results were expressed in µg of GAG/mg of tissue. Assays were performed three times and every time in triplicate.

HS or CS quantification

The DMMB method allows determination of the CS content after HS elimination from samples by nitrous acid treatment as previously described [1]. Briefly, 100 µL of sample, spiked samples, or standards were mixed with 100 µL sodium nitrite (5%) and 100 µL acetic acid (33%). Standards, samples, or spiked samples were gently shaken and kept at rt for 1h. Then, 100 µL of ammonium sulfamate (12.5%) were added and the reaction mixture was shaken for further 5 min. Remaining sulfated GAGs in samples were quantified in 100 µL of reaction mixture by following the DMMB protocol as described above. A calibration curve was constructed by preparing mixtures or known amounts of CS-A and HS treated in the same way.

RESULTS

Optimisation of DNA digestion on PK treated samples

DNase sample treatment was accomplished by adding 7.5 units (U) of the enzyme per 25 mg of brain tissue. To confirm that this DNase I activity can effectively complete DNA digestion in brain samples, DNAse treated samples were analyzed by agarose gel electrophoresis. Results show that, under the assayed conditions, 7.5 U of DNAse totally digested DNA in 25 mg/mL suspensions of brain wet tissue, but not on freeze-dried tissue (S1 File. Fig. 1).

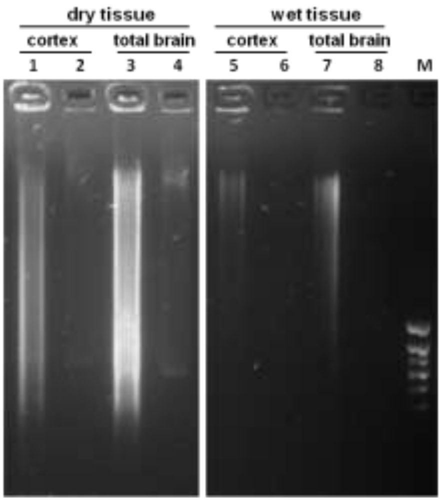

S1 File. Fig. 1. DNA digestion in GAG samples is completed in wet tissue. Agarose (1%) gel was used to assess DNA digestion in brain samples to be used for GAGs quantification. Lane **1**: Freeze-dried cortex treated with PK. Lane **2**: Freeze-dried cortex treated with PK and DNase. Lane **3**: Freeze-dried total brain treated with PK. Lane **4**: Freeze-dried total brain treated with PK and DNase. Lane **5**: Wet cortex treated with PK. Lane **6**: Wet cortex treated with PK and DNase. Lane **7**: Wet total brain treated with PK. Lane **8**: Wet total brain treated with PK and DNase. Lane **M**: Smart marker (Eurogentec). 7.5 U units of DNase were used to treat brain tissue.

Validation of GAG extraction and quantification method

Linearity

Linearity was assessed in freeze-dried and wet tissue samples containing sulfated GAGs at basal levels measured as follows (basal GAGs contents): freeze-dried cortex, 33.9 µg/mL; freeze-dried total brain, 3.32 µg/mL; wet cortex, 11.8 µg/mL; and wet total brain, 9.0 µg/mL of GAGs *per* mL of sample. CS-A spiked samples (5, 10, 20 and 30 µg/mL) were used to assess linearity in each tissue type. For the extraction protocol 100 µL of CS-A spiked PK digested samples were used. GAG extraction and quantification was performed twice the same day (Intra-day quantification) and on different days (Inter-day quantification).

Thus, the spiked samples were treated by the complete extraction protocol. Intra-day linearity coefficient was determined by plotting the CS-A amount expected to be found in spiked samples against the obtained CS-A amount effectively measured in the assay. Linearity regression coefficients were as follows: for freeze-dried cortex, $r^2 = 0.9055$; for freeze-dried total brain, $r^2 = 0.9802$; for wet cortex, $r^2 = 0.9928$, and for wet total brain, $r^2 = 0.9815$. Thus,

the best intra-day linearity was obtained from wet brain tissue. For further experiments only, wet tissue extractions were performed. As shown in S1 File. Fig. 2, inter-day linearity for wet cortex was r^2 = 0.9951 and for wet total brain r^2 = 0.9818.

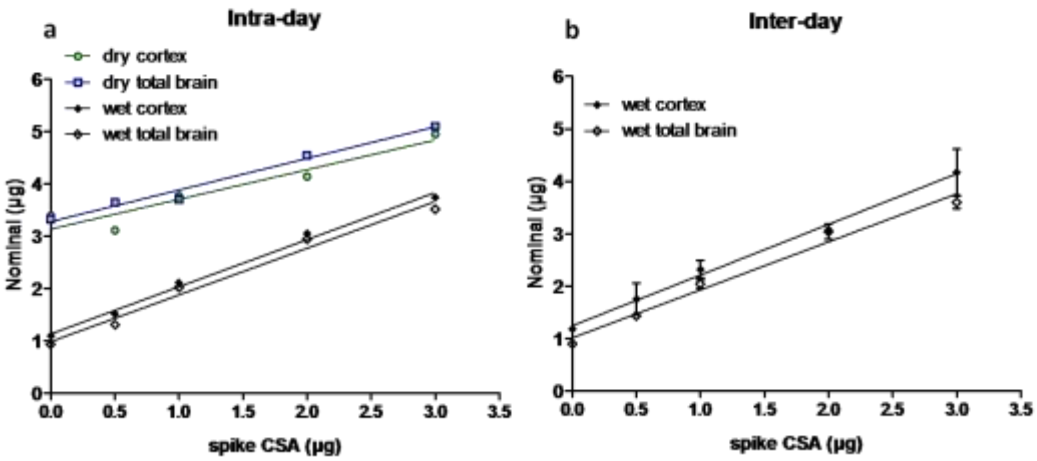

S1 File. Fig. 2. Linearity of the GAGs extraction and quantification method from CS-A spiked samples. PK digested freeze-dried and wet tissue samples were used. For linearity assessment samples were spiked with known amounts of CS-A (0.5 to 30 µg/mL corresponding to 0.5 to 3 µg of CS-A in 100 µL of assayed samples). Intra and inter-day linearity was assessed. **a)** Freeze-dried cortex intra-day linearity (rounds, r^2=0.9055); freeze-dried total brain intra-day linearity (squares, r^2=0.9802), wet cortex intra-day linearity (small diamonds, r^2=0.9928), and total wet brain intra-day linearity (big diamonds, r^2=0.9815). **b)** Wet cortex inter-day linearity (small diamond, r^2=0.9951) and total brain inter-day linearity (big diamonds, r^2=0.9818).

Precision (repeatability).

Precision was defined as the degree of repeatability of the analytical method; it corresponds to the percentage relative standard deviation (coefficient of variation or % CV) analyzed in spiked samples. An excellent precision is often considered for values lower to 10%. Here, intra-day precision of wet tissue (cortex, means=2.3%, total brain, means=2.6%) and inter-day precision of wet tissue (cortex, means=8.2 %, total brain, means=3.4%) were respectively obtained by the analysis of spiked samples on one day and on two different days on CS-A spiked samples (S1 File. Table 1 and 2). These two measurements gave a good repeatability.

Accuracy/Extraction Recovery

Accuracy was defined as the measure of the exactness of the method; it corresponds to the relative percentage of recovery from the theoretical nominal sample concentration by using the spiked samples. Accuracy was ascertained by repetitive analysis (*n*=2, assayed in duplicate) of CS-A spiked samples (S1 File. Table 1 and 2). It was calculated by comparing the DMMB responses (OD) of extracted spiked samples with an extracted or not standard calibration curve. For these samples, extraction recovery for cortex (99.5-101.5%) and from total brain (100.1-101.4%) was expressed as the relative percent of recovery from the nominal sample concentration.

Limits of detection and quantification

The limit of detection (LOD), defined as the lowest amount detectable with a signal to noise ratio of about 3, was 100 ng of GAGs in sample. The limit of quantification (LOQ), defined as the lowest amount quantifiable with a precision of less than 10%, was 400 ng of sulfated GAGs in sample.

Sample stability

GAGs were found to be stable in the final extract kept at -20 °C for at least 1 year. The good stability of extracts under experimental conditions, i.e. during agitation at room temperature, or during the enzymatic digestion conditions, was investigated using controls and standard solutions.

S1 File. Table 1. Validation of the sulfated GAGs extraction and quantification method in wet cortex.

**Sulfated GAGs content in wet cortex**				
	**Nominal µg**	**Found µg**	**Precision %CV**	**% recovery**
Intra-day				
Basal	1.18	1.15	3.7	97.5
Basal + 0.5 µg	1.68	1.53	0.5	91.1
Basal + 1.0 µg	2.18	2.20	3.7	101.0
Basal + 2.0 µg	3.18	3.05	0.1	95.9
Basal + 3.0 µg	4.18	3.86	3.5	92.4
mean			***2.3***	***95.5***
Inter- day				
Basal	1.18	1.18	3.6	100.0
Basal + 0.5 µg	1.68	1.75	17.8	104.2
Basal + 1.0 µg	2.18	2.32	7.4	106.5
Basal + 2.0 µg	3.18	3.08	1.6	97.0
Basal + 3.0 µg	4.18	4.17	10.6	99,9
mean			***8.2***	***101.5***

S1 File. Table 2. Validation of the sulfated GAG extraction and quantification method in wet total brain.

**Sulfated GAGs content in wet total brain**				
	**Nominal µg**	**Found µg**	**Precision %CV**	**% recovery**
Intra-day				
Basal	0.90	0.93	0.6	103.0
Basal + 0.5 µg	1.40	1.42	6.0	101.8
Basal + 1.0 µg	1.90	1.98	2.0	104.2
Basal + 2.0 µg	2.90	2.94	3.3	101.3
Basal + 3.0 µg	3.90	3.52	1.3	90.3
mean			***2.6***	***100.1***
Inter- day				
Basal	0.90	0.90	4.2	100.0
Basal + 0.5 µg	1.40	1.43	0.9	102.4
Basal + 1.0 µg	1.90	2.04	4.0	107.3
Basal + 2.0 µg	2.90	3.04	4.7	104.8
Basal + 3.0 µg	3.90	3.60	3.2	92,4
mean			***3.4***	***101.4***

CONCLUSION

We have developed and validated a procedure for GAGs extraction and quantification from wet brain tissue (cortex or total brain). The method showed excellent linearity, precision and accuracy. It avoids complex steps as chromatographic separations or alcohol precipitation which are time consuming and result in big material lost, with lower linearity and low recoveries (results not shown). The validation was performed by two inter-days assays;

recovery was excellent with a good linearity, precision, and accuracy. This allowed validation and utilisation of the procedure for sulfated GAG quantification in brain.

Reference

1. Barbosa, I., et al., Improved and simple micro assay for sulfated glycosaminoglycans quantification in biological extracts and its use in skin and muscle tissue studies. *Glycobiology*, **2003**. 13(9): p. 647-53.
