# Supplementary material for: Glycosaminoglycans from Alzheimer’s disease hippocampus have altered capacities to bind and regulate growth factors activities and to bind tau
Source: PLoS One. 2019 Jan 4;14(1):e0209573. doi: 10.1371/journal.pone.0209573 (PMC6319808; doi:10.1371/journal.pone.0209573)
Supplement: S1 Table — (DOC) [file pone.0209573.s002.doc]

| **Recombinant proteins/Antibodies** | **Reference** |
| --- | --- |
| Recombinant Human FGF-1 | R&D system 232-FA |
| Recombinant Human FGF-2 | Promokine C-60240 |
| Recombinant Human HB-EGF | R&D system 259-HE/CF |
| Recombinant Human VEGF165 | R&D system 293-VE |
| Recombinant Human BDNF | R&D system 248-BD/CF |
| Recombinant Human PTN | Sigma-Aldrich P5333-50UG |
| Recombinant Human tau | Sigma-Aldrich AG960 |
|  |  |
| Anti-human FGF-1 | R&D system AF232 |
| Anti-human FGF-2 | R&D system AB-33-NA |
| Anti-human HB-EGF | R&D system AF-259-NA |
| Anti-human VEGF165 | R&D system AB-293-NA |
| Anti-human BDNF | R&D system AF248 |
| Anti-human PTN | R&D system AF-252-PB |
| Peroxidase-conjugated AffiniPure Rabbit anti-Goat IgG | Jackson ImmunoResearch 305-035-045 |
| Peroxidase-conjugated AffiniPure Goat anti-Rabbit IgG | Jackson ImmunoResearch 111-035-144 |
| Peroxidase-conjugated AffiniPure Rabbit anti-Chicken IgY | Jackson ImmunoResearch 303-035-003 |
| Anti-heparan sulfate (clone F58-10E4) | AMS Biotechnology 370255-1 |
| Alexa Fluor 555 | Invitrogen A31570 |
| CY3 | Sigma-Aldrich C2181 |
| DAPI | Thermofisher Scientific D1306 |

**S1 Table. List of recombinant proteins and antibodies**
