# Supplementary material for: Glycosaminoglycans from Alzheimer’s disease hippocampus have altered capacities to bind and regulate growth factors activities and to bind tau
Source: PLoS One. 2019 Jan 4;14(1):e0209573. doi: 10.1371/journal.pone.0209573 (PMC6319808; doi:10.1371/journal.pone.0209573)
Supplement: S2 Table — (DOC) [file pone.0209573.s003.doc]

**S2 Table. List of oligonucleotides for real time qPCR**

| Gene name | Accession number | | Oligonucleotide  sequences (sense) | Oligonucleotide  sequences (anti-sense) |
| --- | --- | --- | --- | --- |
| *NDST-1* | NM_001543 | GGAAGTGTGTCCGTGGTTCT | | CCCTGGTAACTGTGCTCCAT |
| *NDST-2* | NM_003635.3 | CTCCAGTTGTGGAAGGTGGT | | CTTAGGGCTGGTGGACACAT |
| *NDST-3* | NM_004784 | CGACCTCCAACACCTACCAT | | TAGGACTGTGGGGTCTGTCC |
| *NDST-4* | NM_022569 | GCAACGGTGATTCAGGATCT | | TGTGCAGCCAAAAGTTCAAG |
| *HS2ST* | NM_012262 | CGAAGTCCGAGAAATTGAGC | | AATGAAGTGCTTGCCGTTTT |
| *HS3ST1* | NM_005114 | ACCACATGCAGAAGCACAAG | | TTGAGGGCCTTGTAGTCCAC |
| *HS3ST2* | NM_006043 | GGAACCCCACTTCTTTGACA | | GTCGAGGAGCCTCTTGAGTG |
| *HS3ST3A1* | NM_006042 | ACGCCCAGTTACTTCGTCAC | | GAACGTCAAGCTCTCGAAGG |
| *HS3ST3B1* | NM_006041 | ACGCCCAGTTACTTCGTCAC | | TCTGCGTGTAGTCCGAGATG |
| *HS3ST4* | NM_006040 | AAGAGCAAAGGTCGGACTCA | | ACCCTCTTCCTGTTCCCACT |
| *HS3ST5* | NM_153612.3 | GCTAGAGGGGAAGGAGAGGA | | CCATCGACGACATGAAATTG |
| *HS3ST6* | NM_001009606.2 | CTGTCCCACTTCCTGTTCGT | | CCTTGGTGGCGTTGAAGTAG |
| *HS6ST1* | NM_004807 | GGCCCTTCATGCAGTACAAT | | TACAGCTGCATGTCCAGGTC |
| *HS6ST2VarL* | NM_001077188 | CGGGGTTCTCCAAACACTAA | | GTCTCGGAGGATGGTGATGT |
| *HS6ST2VarS* | NM_147175 | AGGCTCCTTCAGACCCATTT | | TCGGATTTGGGTTCTGACTC |
| *HS6ST3* | NM_153456 | CATCTCCCCCTTCACACAGT | | CTCGTAAAGCTGCATGTCCA |
| *GLCE* | NM_015554 | GGAAGTGTGTCCGTGGTTCT | | CCCTGGTAACTGTGCTCCAT |
| *HPSE* | NM_001098540 | ATCAATGGGTCGCAGTTAGG | | AGGCTGACCAACATCAGGAC |
| *TUBA1A* | NM_006009.2 | GCAACAACCTCTCCTCTTCG | | GAATCATCTCCTCCCCCAAT |
| *TBP* | NM_003194.4 | TGCACAGGAGCCAAGAGTGA | | CACATCACAGCTCCCCACCA |
