# Supplementary figures and images for: Glycosaminoglycans from Alzheimer’s disease hippocampus have altered capacities to bind and regulate growth factors activities and to bind tau
Source: PLoS One. 2019 Jan 4;14(1):e0209573. doi: 10.1371/journal.pone.0209573 (PMC6319808; doi:10.1371/journal.pone.0209573)

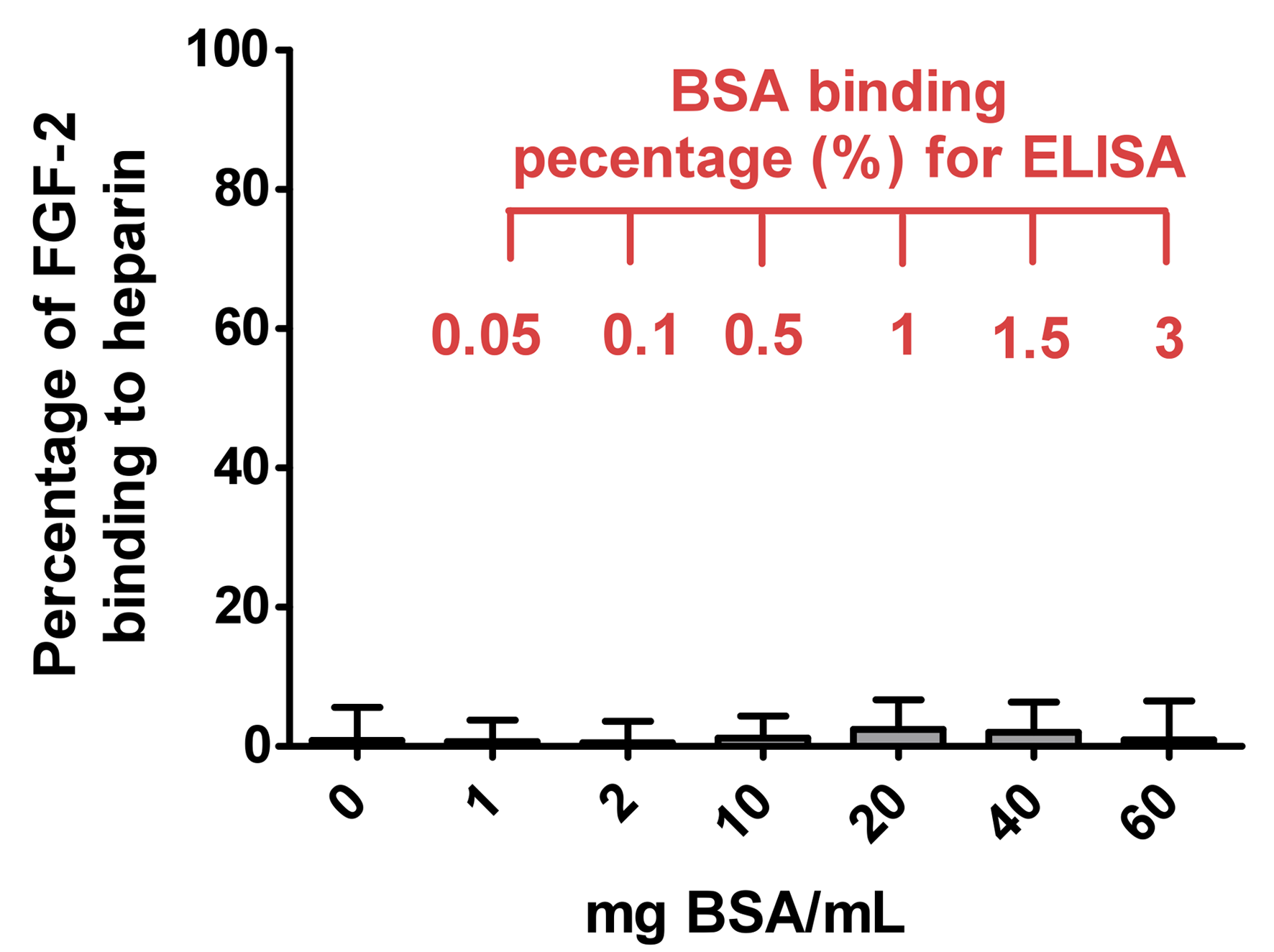

Supplement: S1 Fig — (TIF) [file pone.0209573.s004.tif]

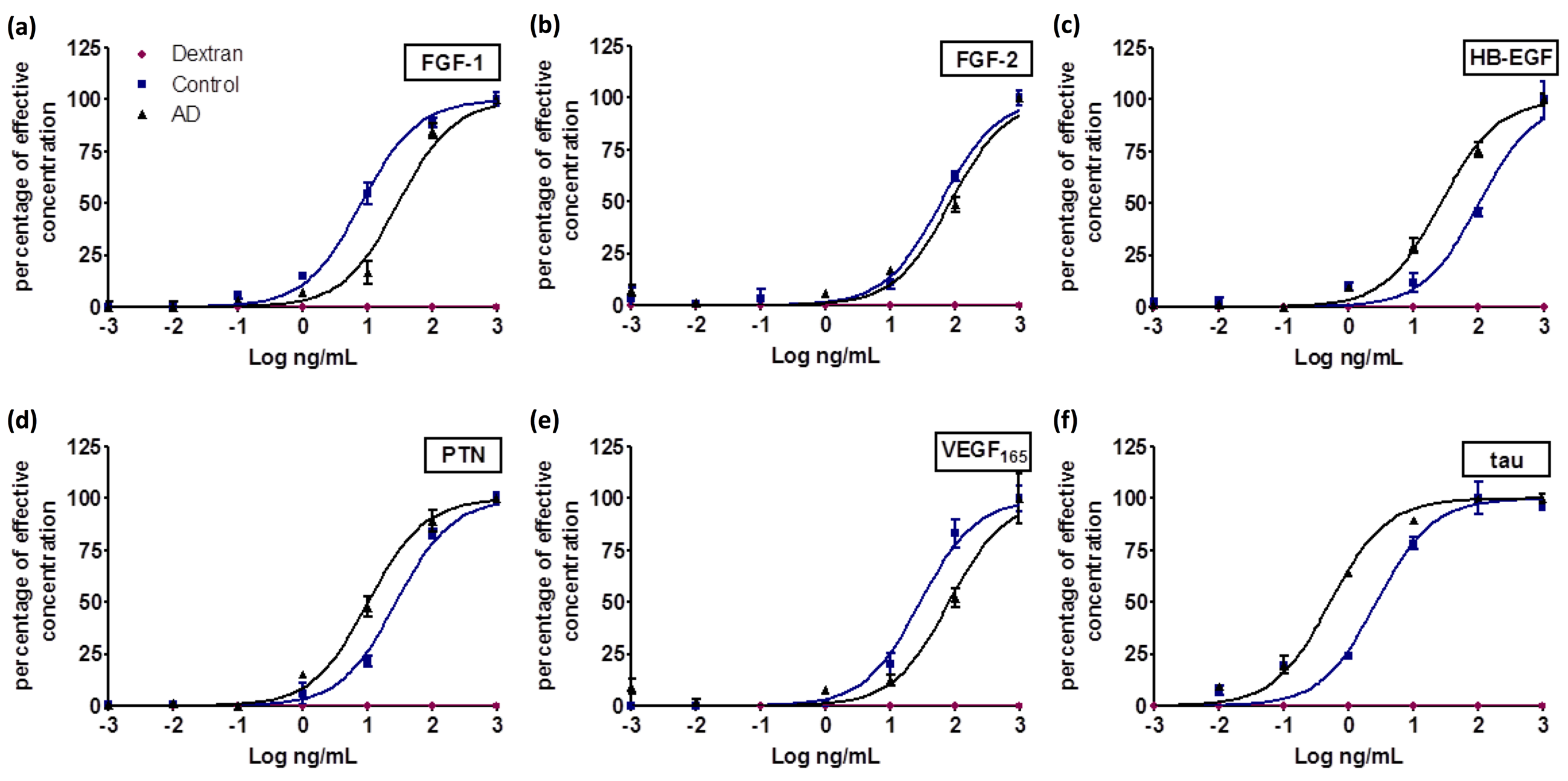

Supplement: S2 Fig — (TIF) [file pone.0209573.s005.tif]
